# Janus kinase 1/2 inhibition with baricitinib in the treatment of juvenile dermatomyositis

**DOI:** 10.1093/brain/awz005

**Published:** 2019-02-01

**Authors:** Charalampia Papadopoulou, Ying Hong, Ebun Omoyinmi, Paul A Brogan, Despina Eleftheriou

**Affiliations:** 1 Infection, Inflammation and Rheumatology Section, UCL Great Ormond Street Institute of Child Health, London, UK; 2 Arthritis Research UK Centre for Adolescent Rheumatology, London, UK

Sir,

We read with interest the recent article by Ladislau and colleagues reporting on the pathogenicity of type I interferon (IFN) *in vitro* and evaluating the efficacy of type I interferon pathway blockade for therapeutic purposes in adults with dermatomyositis (Ladislau *et al.*, 2018). The authors demonstrated that activation of type I IFN in differentiating myoblasts abolished myotube formation with reduced myogenin expression while in differentiated myotubes, they observed a reduction in surface area and an upregulation of atrophy-associated genes (Ladislau *et al.*, 2018). *In vitro* endothelial cells exposure to type I interferon disrupted vascular network organization (Ladislau *et al.*, 2018). All the pathogenic effects observed *in vitro* were abolished by ruxolitinib an orally administered Janus Kinase (JAK) inhibitor. Finally, four adults with refractory dermatomyositis patients were treated with ruxolitinib and improvement ensued in skin lesions, muscle weakness and a reduced serum type I IFN levels and IFN-inducible genes scores (Ladislau *et al.*, 2018). The authors proposed JAK inhibition as a mechanism-based treatment for dermatomyositis, a finding that is relevant for the design of future clinical trials targeting dermatomyositis.

Juvenile dermatomyositis (JDM) is a multi-systemic autoimmune disease characterized predominantly by progressive proximal muscle weakness and pathognomonic skin rashes (Papadopoulou and Wedderburn, 2017). Even though the overall prognosis of JDM has improved significantly in recent years, the long-term outcome of treated JDM differs substantially from patient to patient, and the disease can be life-threatening in some cases (Papadopoulou and Wedderburn, 2017). Similar to adult onset dermatomyositis, it is now increasingly recognized that IFNs play a central role to the pathogenesis of JDM and are important drivers of JDM vasculopathy (Baechler *et al.*, 2011). The recent development of small molecules that inhibit JAKs and reduce type I and type II IFN-induced STAT1 phosphorylation (p-STAT1) has already impacted favourably on the treatment of patients with monogenic intereferonopathies (Sanchez *et al.*, 2018). The report by Ladislau *et al.* now suggests that JAK inhibition may also be of relevance to the treatment of non-genetic IFN-mediated diseases such as adult dermatomyositis (Ladislau *et al.*, 2018). Similarly, JAK inhibition may provide means of targeted therapy for JDM to improve outcomes for children with this rare condition. We now describe for the first time the case of an 11-year-old with refractory to multiple anti-inflammatory therapies JDM who responded to treatment with baricitinib (a JAK 1/2 inhibitor).

The following standardized measures were used to assess JDM disease activity and response to treatment in this case (Ruperto *et al.*, 2010; Campanilho-Marques *et al.*, 2016): (i) muscle strength was assessed using the Childhood Myositis Assessment Scale (CMAS, range 0–52) and the Manual Muscle Testing (MMT) scale (range 0–80); (iii) the physician’s global assessment of the patient’s overall disease activity was indicated on a visual analogue scale (physicians VAS, range 0–10); (iv) skin disease activity was assessed using the modified skin Disease Activity Score (DAS, range 0–5); (v) the parent’s global assessment of the child’s overall well‐being and pain was indicated on a VAS (range 0–10, patient VAS and pain VAS); and (vi) functional ability was assessed using the Child Health Assessment Questionnaire (CHAQ, score 0–3). See Supplementary material for detailed methods of genetic sequencing and assessment of endothelial injury and IFN pathway biomarkers (Clarke *et al.*, 2010; Omoyinmi *et al.*, 2017).

Our patient was a Caucasian male of non-consanguineous descent who was diagnosed with JDM at the age of 2.5 years having presented with typical clinical findings of severe proximal muscle weakness (CMAS of 18/52; MMT-8 of 56/80) and characteristic heliotrope rash, Gottron’s papules over the small and large joints of his hands (skin DAS of 4/5). He had raised creatine phosphokinase (CPK = 403 U/l, reference range 6–330 U/l) and lactate dehydrogenase (LDH = 1509 U/l, reference range 450–770 U/l); modestly elevated erythrocyte sedimentation rate (ESR = 18 mm/h, reference range < 10 mm/h) and normal C-reactive protein (CRP = 3 mg/l, reference range < 20 mg/l). His disease was complicated by pharyngeal involvement; but there was no evidence of interstitial lung disease, gastrointestinal or cardiac involvement. Myositis specific autoantibody detection showed positive anti-TIF1γ and anti-Ro-52 antibodies; antinuclear antibodies were positive with a titre 1:640, negative for dsDNA antibodies, complement function studies were normal and C1q, C3 and C4 levels were all within normal limits. He was initially treated with corticosteroids (30 mg/kg of intravenous methylprednisolone over 3 days followed by 2–3 mg/kg/day with intention to wean over 4–5 months) and subcutaneous methotrexate (15 mg/m^2^ weekly). Over the next 6 months he developed extensive ulcerative skin disease (modified skin DAS range 5/5) at which point intravenous cyclophosphamide (500–750 mg/m^2^ given every 2–3 weeks to a total of six doses) treatment was initiated with some improvement in CMAS 44/52 and modified skin DAS 3/5. Persistence of cutaneous disease over time and development of calcinosis over his elbow joints, right ear lobe required sequential treatment with azathioprine, mycophenolate mofetil, infliximab, adalimumab, rituximab, tacrolimus and ciclosporin, intravenous immunoglobulin (IVIG) over the next 7 years. He remained symptomatic and steroid dependent (2 mg/kg/day of prednisolone). Genetic testing was performed using version 2 (VIP2) of our recently developed targeted gene panel for Vasculitis and AutoInflammation Panel (VIP), which contains 166 genes, to exclude known monogenic interferonopathies that may mimic JDM, such as the proteasome associated autoinflammatory diseases (Omoyinmi *et al.*, 2017). No pathogenic class 4 or 5 variants were identified.

Treatment with baricitinib (a selective JAK1/JAK2 inhibitor) was initiated at age 11.5 years because of ongoing severe skin disease activity and progressive calcinosis (expanded access protocol NCT01724580). He underwent dose escalation until he reached optimal tolerated treatment doses adjusted to his weight and renal function (6 mg twice a day). Clinical symptoms improved upon treatment with baricitinib at this dose with CMAS improving from 46/52 at time of start of therapy to 50/52 at 6 months of treatment; MMT-8 improving from 59/80 at baseline to 70/80 at 6 months; modified skin DAS 5/5 at baseline to 1/5 at 6 months; physician’s VAS from 4.3/10 to 1.5/10 (Supplementary Fig. 1). CPK remained stable ranging between 70 and 109 U/l. The median corticosteroid dose was reduced from a prednisone equivalent dose of 1.7 mg/kg/day at baseline to 0.3 mg/kg/day at 6 months. In addition, the following parameters also improved: CHAQ 1.75 to 0.125; pain VAS from 6.3/10 to 2.1/10; parental VAS from 4/10 to 2/10. No new calcinotic lesions were observed. There were no significant adverse events reported; specifically, no BK or JC viraemia noted or significant cytopaenias.

At 12 months of treatment he stopped taking all his medication against medical advice and this led to a significant flare of his symptoms within 6 weeks with significant deterioration of his skin rash (modified skin DAS 5/5), worsening myalgia and arthralgia, CMAS decrease to 46/52 and MMT-8 to 59/80, physician’s VAS 6/10 (Supplementary Fig. 1), CPK elevated at 426 U/l. Reintroduction of treatment with baricitinib again resulted in clinical improvement observed within 2 weeks: CMAS 52/52, MMT-8 78/80, modified skin DAS 1/5, physician’s VAS 1/10, CPK 155 IU/l at time of latest follow-up at 18 months of treatment and allowed re-tapering of corticosteroids down to 0.25 mg/kg/day of prednisone from 0.3 mg/kg/day (Fig. 1A and B). During treatment with baricitinib our patient’s weight changed from 60.4 kg (91st centile for age) to 77.4 kg (99th centile for age); and height from 148.6 cm (91th centile) to 154. 8 cm (99th centile for age) at time of latest follow-up.

**Figure 1:**
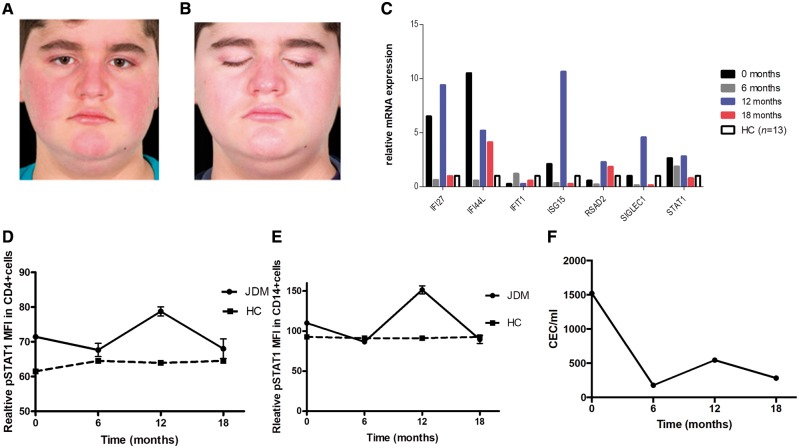
**Clinical and interferon (IFN) biomarker response in a patient with JDM treated with janus kinase (JAK) 1/2 inhibitor (baricitinib).** (**A** and **B**) Improvement in facial skin rash in an 11-year-old male patient with JDM treated with baricitinib. (**C**) IFN induced gene expression at baseline, 6 months, 12 months (flare) and 18 months after starting baricitinib, compared to healthy controls (HC, *n* = 13). (**D**) Signal transducer and activator of transcription 1 (STAT1) phosphorylation in CD4+ cells and (**E**) in CD14+ cells assessed with flow cytometry is shown at time of starting baricitinib treatment baseline (t = 0 months), 6 months, 12 months (flare) and 18 months compared to healthy controls (*n* = 3). (**F**) Circulating endothelial cells (CECs) were measured with immunomagnetic bead extraction at baseline before treatment with baricitinib was started and at 6 months, 12 months (flare) and 18 months after starting treatment. Results are expressed as median and range. MFI = median fluorescence intensity.

Biomarkers of IFN signalling, and the type 1 IFN induced gene expression that were modestly elevated at time of start of treatment (note patient was receiving high doses of corticosteroids at that time point) significantly decreased during treatment with baricitinib at 6 months and 18 months with upregulation at 12 months when all treatment had been stopped due to lack of compliance (Fig. 1C–E). We measured STAT1 phosphorylation to assess type I IFN receptor responsiveness during baricitinib treatment; the STAT1 phosphorylation in CD4+, CD8+ and CD14+ cells was reduced to levels measured in healthy controls at 18 months of latest follow up (Fig. 1D, E and Supplementary Fig. 1). The same trend was observed when STAT1 phosphorylation was measured following stimulation with IFN-α (data not shown). We additionally assessed circulating endothelial cells (CEC) as biomarkers of endothelial injury relating to the vasculopathy of JDM. CEC declined over 18 months of treatment from 1560 cells/ml at diagnosis to 280 cells/ml (healthy control levels range 20–50 cells/ml) (Clarke *et al.*, 2010).

In line with the findings from Ladislau *et al.* (2018) we have now demonstrated clinical improvement in a child with refractory JDM following treatment with baricitinib. We also show that this response was accompanied by downregulation of IFN gene responses and improvement in levels of endothelial injury biomarkers. Deterioration in clinical features and upregulation of IFN pathway related biomarkers during the time of treatment discontinuation and subsequent improvement on reintroduction of treatment further provides clinical and biomarker evidence to support the efficacy of this therapeutic approach in this patient with JDM. We emphasize that our case had very severe disease for years and therefore the potential for complete reversibility of his symptoms remains uncertain. Nevertheless, for the first time in 7 years we were able to significantly reduce his corticosteroid therapy, alter the progression of calcification and document improvement in his symptoms. We remain encouraged by the trend of the IFN pathway biomarkers and CEC, although we have not yet achieved complete clinical remission with baricitinib. Further prospective studies are now needed to explore the potentially safety and efficacy of JAK inhibition in JDM, not only for refractory cases such as the case described herein but also as first line primary therapy.

## Data availability

The authors confirm that the data supporting the findings of this study are available within the article and its Supplementary material.

## Supplementary Material

Supplementary Material
