# Supplementary material for: Janus kinase 1/2 inhibition with baricitinib in the treatment of juvenile dermatomyositis
Source: Brain. 2019 Feb 1;142(3):e8. doi: 10.1093/brain/awz005 (PMC6391598; doi:10.1093/brain/awz005)
Supplement: Supplementary Material [file awz005_supplementary_material.pdf]

Supplementary material

Supplementary methods:

We obtained written informed consent from all the family and controls (ethics approval 08H071382, 11/LO/0330 and MREC/1/3/22) who participated in this study**.**

IFN stimulated gene RNA expression in total blood

Whole blood was collected into PAXgene tubes. We used a PreAnalytix RNA isolation kit to extract total RNA and RNA concentration was assessed with a Nanodrop (FLUOstar Omega, Labtech). We performed quantitative reverse transcription polymerase chain reaction (qPCR) analysis using the iTaq Universal SYBR Green Supermix (172-5121, Bio-Rad), and cDNA derived from 400 ng total RNA. Using Qiagen Quantitec primers for IFI27 (HS_IFI27_1_SG), IFI44L (HS_IFI44L_1_SG), IFIT1 (HS_IFIT1_1_SG), ISG15 (HS_ISG15_1_SG), RSAD2 (HS_RSAD2_1_SG), SIGLEC1 (HS_ISG15_1_SG), CXCL10 (HS_CXCL10_1_SG), STAT1 (Sigma Forward primer: TGCTTGGATCAGCTGCAGAA; Reverse primer: CCGAACTTGCTGCAGACTCT). The relative abundance of each of these target transcripts was normalized to the expression level of HPRT1 (HS_HPRT1_1_SG), assessed using the CFX Maestro software.

STAT-1 phosphorylation

Peripheral blood mononuclear cells (PBMCs) were isolated from blood collected into Falcon tubes (Thermo Fisher Scientific) containing 35 U preservative-free heparin (CP Pharmaceuticals) per 50 ml. Blood was the diluted with the equal volume of RPM1640 medium and overlaid on an equal volume of Ficoll-Paque reagent, and then centrifuged 800 g

for 10 min with the brake off. The PBMC layer was taken, washed and resuspended in warm RPMI with 10% FCS. PBMC were stimulated with 5 ng/ml of IFN-α for 30 minutes. Cells were fixed using BD Cell Fix Buffer (10 min at 37°C) and then permeabilized using BD PermIII Buffer (30min at 4°C). Cells were stained with PE-anti-STAT1 (BD Bioscience, cat: 612564, pY701, 1:50) and other cell surface markers BV711-CD4 (Biolegend, cat: 317440, OKT4, 1:100), BV605-CD14 (Biolegend, cat: 301833, M5E2, 1:100) and BV510-CD8a (Biolegend, cat: 301048, RPA-T8, 1:100) for 1h at room temperature protected from light. Flow cytometry analysis was performed on a BD LSRII flow cytometer. Results were analyzed using FlowJo v10.4.2.

Circulating endothelial cells

Circulating endothelial cells (CECs) were identified with CD146-immunomagnetic bead extraction based on an international consensus standardised protocol (Clarke *et al*, 2010). Venous blood (1 ml) collected into tubes containing EDTA was mixed with buffer (1 ml of phosphate buffered saline containing 0.1% bovine serum albumin and 0.6% sodium citrate) and 20 μl of Fc receptor–blocking reagent (Miltenyi Biotec) and incubated for 5 minutes at room temperature. Fifty microliters of a preparation of anti-CD146-coated immunomagnetic beads (clone S-endo-1; BioCytex and Dynal Biotech) was added, and the sample was incubated at 4°C for 30 minutes, with rotation. Bead-bound cells were separated using a magnet (MPC-L; Dynal Biotech) and washed 3 times with buffer. Cells were then resuspended in 100 μl of buffer containing 10 μl of a 2-mg/ml preparation of FITC-labeled Ulex europapeus lectin (Sigma-Aldrich) and incubated for 1 hour at room temperature in the dark. CECs in the sample were counted using a Nageotte chamber on a fluorescence. CECs were defined as Ulex bright cells that were >10 μm in size, with >5 magnetic beads attached.

Vasculitis and Inflammation Panel (VIP) targeted genetic sequencing

Detailed description of the development and validation of this panel and list of genes included in the panel has been described elsewhere in more detail (Omoyinmi *et al*, 2017). In brief the capture of targeted genes/regions was performed using the Agilent QXT Target Enrichment system according to the manufacturer's protocol (Version B.2, October 2014) for Illumina sequencing. Briefly, genomic DNA was sheared by enzyme fragmentation, and ligated with SureSelect Adaptor Oligo Mix. Fragment size was assessed using the TapeStation 2100 Bioanalyzer (Agilent Technologies). The adaptor ligated libraries were then amplified and hybridized to our customized SureSelect panel. Captured libraries were indexed (barcoded), pooled and sequenced as multiplex of 16 samples on the benchtop next generation Illumina MiSeq sequencer in 150bp paired-end mode according to the standard protocol for this platform. Read alignment, variant calling, and annotation were performed using bioinformatics pipelines. Identified variants were classified according to pathogenicity, evaluated for coverage and visually inspected using the Integrative Genomics Viewer (Broad Institute).

Myositis specific autoantibody detection

Detection of myositis specific autoantibodies was performed through the routine clinical lab at Great Ormond Street Hospital NHS Foundation Trust using a combination of immunoprecipitation and ELISA (Tansley *et al.*, 2017). Screening included testing for the antibodies against the following: TIF1γ, OJ, EJ, PL12, PL7, SRP, Jo-1, Pm-Scl75, Pm-Sc1l100, Ku, SAE, NXP-2, MDA5, Mi-2, Ro-52.

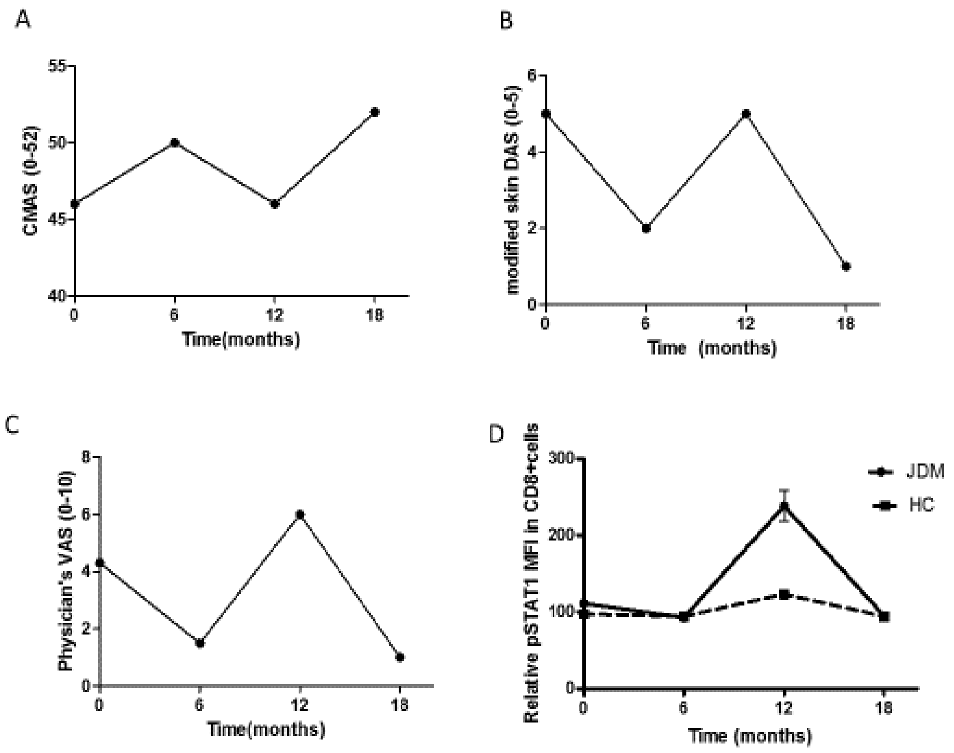

Supplementary Figure 1: A-C Changes in Childhood Myositis Assessment Scale (CMAS), modified skin Disease Activity Score (DAS, range 0-5) and Physician's visual Analogue Scale (VAS, range 0-10) over time for a patient with Juvenile dermatomyositis (JDM) treated with baricitinib. **Supplementary Figure 1D** Signal transducer and activator of transcription 1 (STAT1) phosphorylation in CD8+cells is shown at baseline (t=0 months) at time of starting baricitinib treatment, 6 months, 12 months (flare) and 18 months compared to healthy controls (n=3). MFI=median fluorescence intensity.
